# Maternal high fat diet exposure modifies amniotic fluid metabolites and expands group 3 innate lymphoid cells dependent on the maternal microbiome and MyD88-signaling

**DOI:** 10.3389/fimmu.2024.1439804

**Published:** 2024-11-18

**Authors:** Xinying Niu, Dongmei Lu, Sana Jaleel, Suzette N. Palmer, Mala Mahendroo, Xiaowei Zhan, Julie Mirpuri

**Affiliations:** ^1^ Division of Neonatal-Perinatal Medicine, Department of Pediatrics, University of Texas Southwestern, Dallas, TX, United States; ^2^ Department of Biomedical Engineering, University of Texas Southwestern, Dallas, TX, United States; ^3^ School of Public Health, Center for Genetics of Host Defense, University of Texas Southwestern, Dallas, TX, United States; ^4^ Department of Obstetrics and Gynecology, University of Texas Southwestern, Dallas, TX, United States

**Keywords:** maternal high fat diet, metabolites, intestinal inflammation, innate immunity, fetal, neonatal, amniotic fluid

## Abstract

**Background:**

Maternal high fat diet (mHFD) exposure expands IL-17 producing group 3 innate lymphoid cells (IL17^+ve^ ILC3) in the small intestine of neonatal murine offspring and increases their susceptibility to intestinal inflammation. How mHFD modulates innate immunity in the fetal offspring remains unclear.

**Methods:**

Dams were exposed to 60% high fat diet or maintained on regular diet (RD) prior to and during mating. Amniotic fluid (AF) was collected during mid-pregnancy and metabolites examined by global non-targeted mass spectrometry in conventional wild-type (WT) and germ-free pregnant dams. Offspring were delivered by C-section or vaginally and fecal contents examined for major bacterial phyla and small intestinal lamina propria cells (LP) by flow cytometry. Susceptibility to intestinal inflammation was determined using a lipopolysaccharide and platelet-activating factor model (LPS/PAF) in WT, germ-free and MyD88 deficient offspring. Neonatal germ-free pups were exposed to HFD or RD AF by gavage and LP examined by flow cytometry.

**Results:**

We identified differentially produced metabolites in mHFD AF when compared to RD AF in conventionally raised mice, with no difference seen in germ-free mice. C-section delivery maintained the mHFD phenotype of expansion of IL17^+ve^ ILC3 and increased susceptibility to inflammation in neonatal offspring. In addition, mHFD offspring had expansion of IL17^+ve^ ILC3 at birth and 2 weeks of life, which was not seen in germ-free and MyD88 KO mice exposed to mHFD. Germ-free and MyD88 KO mice were protected from mHFD induced LPS/PAF injury and IL17^+ve^ ILC3 expansion, demonstrating that the maternal microbiome and MyD88 are prenatally necessary for the expansion of IL17^+ve^ ILC3 in mHFD offspring. Furthermore, introduction of mHFD AF to neonatal germ-free pups by gavage was sufficient to expand IL17^+ve^ ILC3 in the small intestine.

**Conclusion:**

Our findings indicate that mHFD interacts with the maternal microbiome to modify AF metabolites and signal via MyD88 to expand IL17^+ve^ ILC3 in murine neonatal offspring.

## Introduction

There is increasing evidence that exposure to maternal high fat diet (mHFD) affects offspring outcomes (1). This includes changes to offspring innate mucosal immunity (2–4), reproduction (5, 6), modification of metabolism (7), molecular alterations to the brain and behavior (8, 9) and increased adiposity (7, 10). We have previously shown that mHFD exposure expands IL-17 producing group 3 innate lymphoid cells (IL17^+ve^ ILC3) in the small intestine of neonatal murine offspring and increases susceptibility to intestinal inflammation (2). ILC3 are emerging as a critical component of neonatal immunity, with potential critical roles in development of necrotizing enterocolitis, late onset sepsis and bronchopulmonary dysplasia (11). The mechanisms of mHFD mediated expansion of IL17^+ve^ ILC3 in the fetus and offspring have not been fully examined to date.

Worldwide rates of obesity have been rising (12) and over 40% of women of childbearing age in the United States are obese (13). Maternal obesity is an important public health challenge with health consequences for offspring, including increasing the propensity to developing chronic conditions (14). While obesity is a multifactorial condition, diet is an important contributor. Maternal diet can modify the maternal microbiome (15) and there is increasing evidence of maternal diet mediated effects on fetal and neonatal immune development (16–18).

ILC3 can produce both IL-17 and IL-22, with mHFD exposure specifically expanding mucosal IL17^+ve^ ILC3 in offspring, partially dependent on a Firmicutes-rich neonatal microbiome (2). MyD88 is a critical sensor for microbial signals and has been implicated in diet-induced metabolic disorders (19–21). Amniotic fluid (AF) is produced early in gestation from ultrafiltration of maternal serum. AF is known to contain metabolites and is constantly swallowed by the fetus, exposing the intestinal mucosa to these metabolites (22, 23). In the fetus, mucosal IL-22 producing ILC3 require maternal bacterial colonization and exposure to microbial metabolites for expansion (24). The role of the maternal microbiome, MyD88 signaling and amniotic fluid metabolites on fetal and neonatal mHFD induced expansion of IL17^+ve^ ILC3 remains to be investigated.

In this study, we examined the mechanisms of how mHFD exposure expanded IL17^+ve^ ILC3 in neonatal offspring. We revealed through untargeted metabolomics of AF a unique metabolite signature in WT mHFD when compared to RD dams that is absent in AF from germ-free mHFD and RD dams. We further show that expansion of IL17^+ve^ ILC3 in mHFD offspring is independent of the mode of delivery, suggesting a prenatal mechanism. We show that mHFD mucosal IL17^+ve^ ILC3 expansion in neonatal mice is ameliorated in germ-free mice and global MyD88 knockout mice at birth and at 2-weeks-old. Finally, we demonstrate that gavage of mHFD amniotic fluid can independently expand IL17^+ve^ ILC3 in neonatal germ-free mice.

## Methods

### Mice

C57BL/6 mice (WT) were obtained from the University of Texas Southwestern (UTSW) Medical Center Mouse Breeding Core Facility. MyD88 deficient (MyD88^-/-^) mice were originally obtained from the Jackson laboratory and had been breeding in our facility for a minimum of 2 generations prior to the experiments performed. Germ-free (GF) C57BL/6 mice were maintained in gnotobiotic isolators (25) at a core gnotobiotic mouse facility at UTSW Medical Center.

### Diet

Dams were placed on either regular diet or high fat diet. The regular diet (RD) contains 12% fat, 22% protein, and 66% carbohydrate (Envigo, 2918). The high fat diet (HFD) contains 60% fat, 20% protein, and 20% carbohydrates (Research Diets, D12492i). Germ-free regular diet was autoclaved and germ-free HFD was double-irradiated.

### HFD model and breeding

At 4 weeks of age, at the time of weaning, female mice were started on HFD or RD for 4 weeks before breeding. Male mice were maintained on RD until the time of mating. During the mating period which began at 8 weeks of age the females on the HFD continued on the HFD and their mating partner were maintained on the HFD. The females on the RD and their mating partner were maintained on the RD. Breeding continued for a maximum of 16 weeks. Dams were tested for glucose intolerance by 1 hour fasting glucose testing monthly. None of the dams on RD or HFD demonstrated glucose intolerance during this time. HFD dams were heavier compared to RD females but not obese (**Supplementary Figure 1**). All neonatal offspring were examined and not differentiated by sex.

### Cesarean section and cross-fostering experiment

Timed pregnancies were performed on females on either RD or HFD by mating with a male and separating after visualization of a mucous plug, defined as Day 1. At E20, pregnant female mice were euthanized and cesarean section (C-section) was immediately performed, or they were allowed to deliver vaginally. These pregnancies were the dams first pregnancy. Neonatal mice born by C-section were dried and stimulated with blunt forceps and kept warm with a heat lamp. When neonatal mice were vigorous, they were immediately placed with a foster mother on RD with a neonatal litter that was culled.

Nomenclature used for natural and adoptive mothers are as follows: (a) RD/RD, offspring born to RD mothers and cross-fostered to RD mothers until euthanasia; and (b) HFD/RD, offspring born to HFD mothers cross-fostered to RD mothers where they were maintained until euthanasia. Pups born by C-section are designated with a (C).

### Amniotic fluid collection

Timed pregnancies were performed on females on either RD or HFD at their first pregnancy as described above. At E15 amniotic fluid (AF) was collected from individual uterine sacs with no contamination of blood and flash frozen. For unbiased metabolomic profiling by flow injection mass spectrometry, AF samples were thawed on ice for 30 minutes and aliquoted to 20 μl of AF with 180μl of aqueous 80% LCMS-grade methanol (Fisher #A456). This was vortexed for 15 seconds to precipitate protein and incubate at one hour at 4 degrees Celsius. This was then centrifuged at room temperature at 14000g for 15 mins to pellet the precipitate. 100μl of the supernatant was transferred to a fresh microtube and used for metabolite analysis as described below.

### Germ-free gavage of amniotic fluid

For germ-free gavage experiment, RD germ-free (GF) pregnant dams were placed in individual isolators to maintain sterile conditions. After delivery, beginning at 5 days old, GF pups were gavaged with 100μl of either PBS, pooled RD AF (AF^RD^) or pooled HFD AF (AF^HFD^) every other day using a sterile neonatal murine gavage needle in a strictly controlled germ-free isolator facility. AF was normalized by volume between the HFD and RD dams. Neonatal mice were sacrificed and examined at 2 weeks of life by flow cytometry.

### qRT-PCR

Total RNA from whole small intestine was purified using TRIzol reagent and subjected to first-strand cDNA synthesis by using iScript Reverse Transcription Supermix (Bio-Rad). DNA from colonic mucosal fecal contents collected at 2 weeks old was extracted using ZR Fecal DNA miniprep (Zymo Research) and quantified using a NanoDrop 2000c Spectrophotometer (Thermo Fisher Scientific). Quantitative real-time PCR (qRT-PCR) was performed using SsoAdvanced Universal SYBR Green Supermix (Bio-Rad) and the CFX Connect Real-Time system (Bio-Rad) according to the manufacturer’s instructions. For cytokines, data were analyzed by the Ct method with normalization for starting template performed using a housekeeping gene, SRP-14. For bacterial 16S rRNA analysis, samples were normalized to Eubacteria utilizing known-concentration standards. Primer sequences used are as follows: murine SRP-14 5′-AAGTGTCTGTTGAGAGCCACGGAT-3′ and 5′-CTGTCACTGTGCTGGTTTGCTCTT-3′; IL-17 5′-TCCCTCTGTGATCTGGGAA–3′ and 5′–CTCGACCCTGAAAGTGAAGG–3′; TNF-α 5′-CCACCACGCTCTTCTGTCTAC-3′ and 5′-TGGGCTACAGGCTTGTCACT-3′; IL-1β 5′-CCTTCCAGGATGAGGACATGA-3′ and 5′-TGAGTCACAGAGGATGG-GCTC-3′; IL22 5′-CCCATCAGCTCCCACTGC-3′ and 5′-GGCACCACCTCCTGCATATA-3′; IL-10 5′-ATTTGAATTCCCTGGGTGAGAAG-3′ and 5′-CACAGGGGAGAAATCGATGACA-3′. Bacterial primers used are as follows: Eubacteria 5′-ACTCCTACGGGAGGCAGCAGT-3′ and 5′-ATTACCGCGGCTGCTGGC-3′; Enterobacteriaceae 5′-GTGCCAGCMGCCGCGGTAA-3′ and 5′-GCCTCAAGGGCACAACCTCCAAG-3′; Bacteroidetes 5′-GGTTCTGAGAGGAGGTCCC-3′ and 5′-GCTGCCTCCCGTAGGAGT-3′; Firmicutes 5′-GGAGYATGTGGTTTAATTCGAAGCA-3′ and 5′-AGCTGACGACAACCATGCAC-3′.

### Isolation of intestinal lamina propria cells and flow cytometry

Isolation of LP cells was performed as follows and previously described (2). Briefly, the small intestine was removed and opened longitudinally, washed of fecal contents, cut into smaller sections, and subjected to 2 sequential incubations in PBS with 0.5 mM EDTA and 0.2 mM DTT at 37°C with agitation at 220 rpm to remove epithelial cells. The solution was discarded between incubation steps and replaced. The remaining tissue was agitated in PBS and then filtered through a strainer. The tissue was pat dried and minced and placed in the incubator for 30 minutes with gentle agitation at 110 rpm in 0.4 mg/ml collagenase D and 50 mg/ml DNase I at 37°C. The samples were then washed through a strainer (100 μm) and centrifuged at 160g at 4°C. LP cells were then washed with FACS buffer (PBS, 1% EDTA, 1% FBS) and stained with antibody cocktail for 20 minutes at 4°C. The following antibodies were used (all from eBioscience unless otherwise noted): CD45-FITC (30-F11), CD117-APC (180627) (R&D Systems), NKp46–PerCP (29A1.4), CD127-PE-Cy7 (A7R34), CD45-PE-Cy7 (30-F11), CCR6-PE-Cy7 (29-2L17 from Biolegend) and FITC-Lineage (145-2C11; RB6-8C5; RA3-6B2; Ter-119; M1/70 from Biolegend). After extracellular staining, cells were fixed in IC fixation buffer (eBioscience), washed in permeabilization buffer (eBioscience), and stained for intracellular antigens IL-17A-PE (eBio17B7), biotin-conjugated IL-22 (poly 5164, Biolegend), anti–mIL-17A-PerCP (Thr22-ALA158, R&D Systems) and RORγt-PE (AFKJS-9) for 1.5 hours at 4°C. Samples were read on a FACSCanto (BD) and analyzed using FlowJo Software Version 10 (Tree Star).

### Gating for group 3 innate lymphoid cells

Flow cytometry was analyzed using FlowJo 10. Total ILC3 population was gated as CD45^+^,Lin^-^,CD127^+^,CD117^+^,RORγt^+^ cells from all live single cells. IL17A producing ILC3 (IL17^+ve^ ILC3) were gated based on presence of intracellular IL-17A. FMO controls were utilized for gating. The gating strategy is shown in **Supplementary Figure 2**.

### Neonatal LPS/PAF model of intestinal injury

Gut mucosal injury was induced in 2-week-old mouse pups by intraperitoneal administration of lipopolysaccharide (LPS, 1 mg/kg) and platelet-activating factor (PAF, 50 μg/kg) as previously described (26) and outlined briefly here. Neonatal mice were given LPS/PAF intraperitoneally and sacrificed after 2 hours. Distal small intestinal sections were fixed in Carnoy fixative, embedded in paraffin, and stained with hematoxylin and eosin (H&E). Histologic preparations were reviewed on a Leica DM2000 microscope. Images were acquired at 10x and 20x magnification using an Optronics Microfire CCD color camera and PictureFrame 2.0 acquisition software (Optronics). Histological changes were analyzed in a double-blind fashion using a 17-point scale as previously described (26) and as follows. For crypt integrity: 0, normal; 1, irregular crypts; 2, mild crypt loss; 3, severe crypt loss; 4, complete crypt loss with an intact epithelial cell layer; 5, complete loss of crypts and surface epithelium (<10 crypt width); and 6, complete loss of crypts and surface epithelium (>10 crypts). For infiltration of inflammatory cells into the mucosa: 0, normal; 1, mild; 2, modest; and 3, severe. For infiltration of the submucosa: 0, normal; 1, mild; 2, modest; and 3, severe. For infiltration of the muscle: 0, normal; 1, mild; 2, modest; and 3, severe. These scores were added, resulting in a total scoring range of 0 to 15. Three experiments were performed with 4–6 mice in each group unless otherwise stated.

### Metabolomics analysis

Unbiased amniotic fluid metabolomic profiling by flow injection mass spectrometry analysis was performed by General Metabolics. (Boston, MA). Aliquots of the amniotic fluid samples used for MSS were sent to General Metabolics; subjected to methanol extraction; and analyzed by LC-qToF in negative ion mode. Compounds were identified by automated comparison to reference chemical library entries with subsequent visual inspection for quality control. For statistical analyses and data display, any missing values were assumed to be below the limits of detection; these values were imputed with the compound minimum (minimum value imputation). Standard statistical analyses (e.g., Welch’s two sample *t* test) were performed in ArrayStudio (Omicsoft) and Limma R package on log-transformed data; *P* <.05 was considered significant. An estimate of the false discovery rate (*q*-value) was also calculated to take into account the multiple comparisons that normally occur in metabolomics-based studies, with *q* < 0.05 used as an indication of high confidence in a result.

### Statistics

Data were analyzed by 1-way ANOVA with Tukey’s *post hoc* test, 2-way ANOVA, or unpaired 2-tailed Student’s t test, using GraphPad Prism 9. Sample size for flow cytometry, microbiome and cytokine analysis was based on effect size seen in previously published data (2). Data are expressed as mean ± SEM and significance was defined as p < 0.05.

## Results

### Amniotic fluid metabolites are uniquely modified in conventional mHFD dams when compared to RD dams

**Figure 1:**
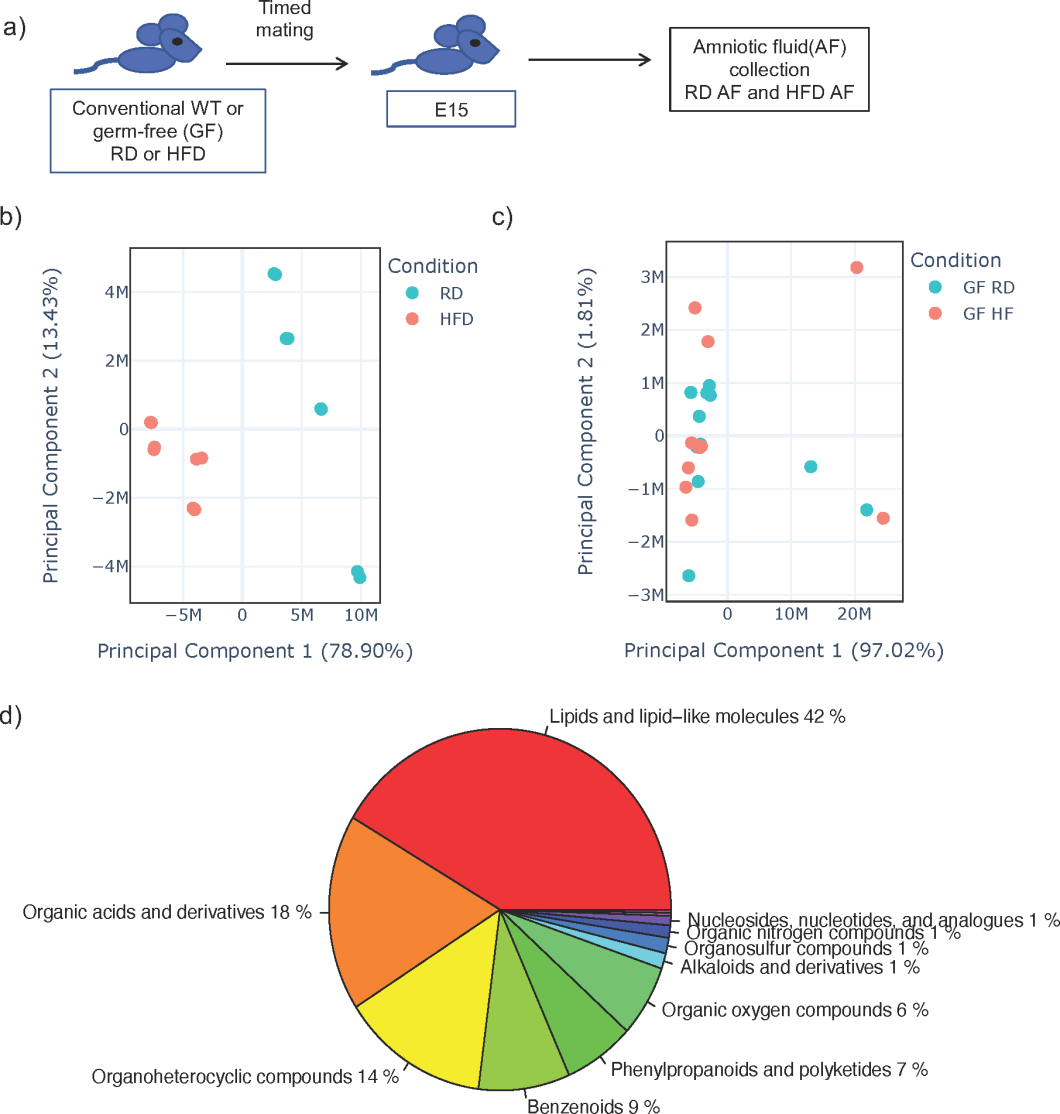
Amniotic fluid metabolites from conventional dams exposed to high fat diet are uniquely clustered when compared to dams on regular diet. **(A)** Experimental design. Timed mating was performed on conventional dams on either regular diet (RD) or high fat diet (HFD). Amniocentesis was performed at E15 of the first pregnancy and amniotic fluid (AF) was sent for untargeted metabolomics by flow injection mass spectrometry. **(B)** Principal component analysis (PCA) plot of leading principal components (PC1 and PC2) of AF from conventional RD and HFD dams demonstrating unique clustering of HFD AF compared to RD AF. **(C)** PCA of AF from germ-free RD and HFD dams demonstrating similar clustering of HFD AF compared to RD AF. Teal dots represent RD AF and red dots represent HFD AF. **(D)** Pie chart of major components of AF metabolites from RD conventional dams. The data shown are representative of AF from 3 dams in all groups with 8 AF samples from individual sacs for conventional RD and 8 from conventional HFD mice and 12 samples from individual sacs for GF RD and 11 samples from individual sacs for GF HFD samples.

AF is produced early in gestation primarily by ultra-filtration of the maternal plasma. The fetus ingests significant amounts of AF during gestation and the intestinal mucosa is consistently exposed to AF metabolites. We hypothesized that AF metabolites can be modified by diet interacting with the maternal microbiome. We performed timed mating of RD and HFD WT conventional and GF dams and collected AF from the first pregnancy in dams at E15 (**Figure 1A**). We analyzed the AF utilizing global untargeted flow injection mass spectrometry. We identified differential metabolite composition in conventional HFD AF when compared to RD AF, as demonstrated by principal component analysis (PCA) (**Figure 1B**) with specific changes in lipids and lipid like molecules. (**Supplementary Figure 3**).

We next examined AF from GF HFD and GF RD mice by global untargeted flow injection mass spectrometry analysis and found no difference in metabolite composition by PCA (**Figure 1C**), strongly suggesting that the maternal microbiome interacts with maternal diet to produce AF metabolites. Overall, from WT and GF mice, we identified over 1000 metabolites in AF and analysis showed that it consisted primarily of lipids (42%), organic acids (18%) and organoheterocyclic compounds (14%) (**Figure 1D**). Comparing metabolites in conventional and germ-free AF, we detected only 29 overlapping metabolites, which is consistent with published studies demonstrating that the majority of metabolites found in the cecum is dependent on both the host and the microbiome (**Supplementary Figure 4**) (27).

### mHFD increased IL17^+ve^ ILC3 and expanded Firmicutes in offspring independent of the mode of delivery

**Figure 2:**
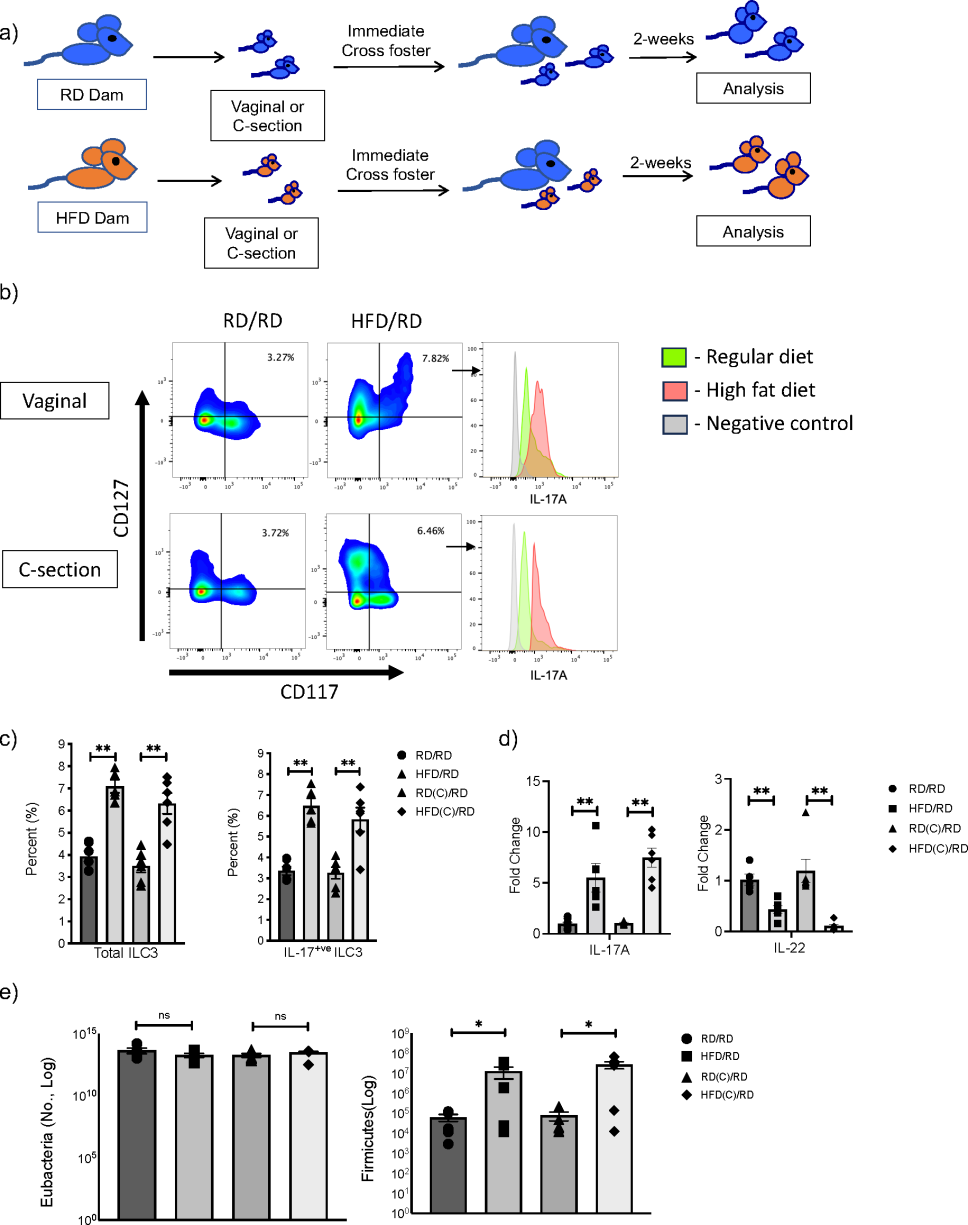
Maternal exposure to high fat diet (HFD) increases Firmicutes and expands IL-17 producing ILC3 in offspring intestine independent of the mode of delivery. **(A)** Experimental design. Timed pregnancies were performed on both regular diet (RD) and HFD dams and offspring were delivered vaginally or by C-section. At birth, offspring were cross-fostered to a RD dam and offspring were examined at 2-weeks-old. **(B)** Representative flow cytometric panels gated for ILC3 and histograms of intracellular markers of IL-17A positive ILC3 from HFD (red) and RD (green) mothers born vaginally (top panel) and by C-section (bottom) compared to control (gray). Offspring showed expansion of ILC3 with maternal HFD exposure and increased IL-17A. **(C)** Flow cytometric quantification of total ILC3 and IL- 17+ ILC3 in RD and HFD offspring born vaginally and by C-section (data consists of 6 samples in each group, 2 experiments for C-section and 3 experiments for vaginal delivery). **(D)** Quantification of IL-17A and IL-22 mRNA in RD and HFD offspring born vaginally and by C-section (n=6-7 mice per group). **(E)** Quantification of Firmicutes in colonic fecal samples at 2 weeks old from RD and HFD offspring born vaginally or via C-section (n= 6 mice per group). Data are depicted as mean +/- SEM with *p < 0.05 and **p < 0.01 according to one-way ANOVA with post-hoc Tukey's test. ns, not significant.

ILC3 are a heterogenous population that can produce the cytokines IL-17A and IL-22 and we have previously demonstrated, utilizing Rag deficient mice, that mHFD exposure specifically increased IL-17^+ve^ ILC3 and expanded Firmicutes in neonatal offspring (2). To determine whether this effect was mediated by exposure to the maternal microbiome during vaginal delivery or a prenatal effect independent of mode of delivery, we delivered offspring born to RD or HFD dams by C-section at E20 or via normal vaginal delivery. We immediately cross-fostered offspring after birth to RD foster mothers and evaluated offspring at 2-weeks-old (**Figure 2A**).

We found that offspring born to HFD mothers and delivered via C-section had an expansion of total ILC3 similar to mHFD offspring delivered vaginally (**Figures 2B, C**), with an increased proportion of IL-17A^+ve^ ILC3. qRT-PCR also confirmed an increase in IL-17 expression in the small intestine of mHFD offspring born both vaginally and via C-section (**Figure 2D**), interestingly we found a decrease in expression of IL-22. When we examined the microbiome in mHFD offspring, we found that there was a similar expansion in Firmicutes in offspring born via C-section when compared to vaginal birth (**Figure 2E**). We found no difference in other major bacterial phyla (**Supplementary Figure 5**).

Combined, this data strongly suggested that the mHFD phenotype was independent of the mode of delivery and a prenatal component was responsible for mediating the expansion of Firmicutes and IL17^+ve^ ILC3 seen in mHFD offspring.

### Germ-free and MyD88 deficient mHFD offspring do not have expansion of ILC3

**Figure 3:**
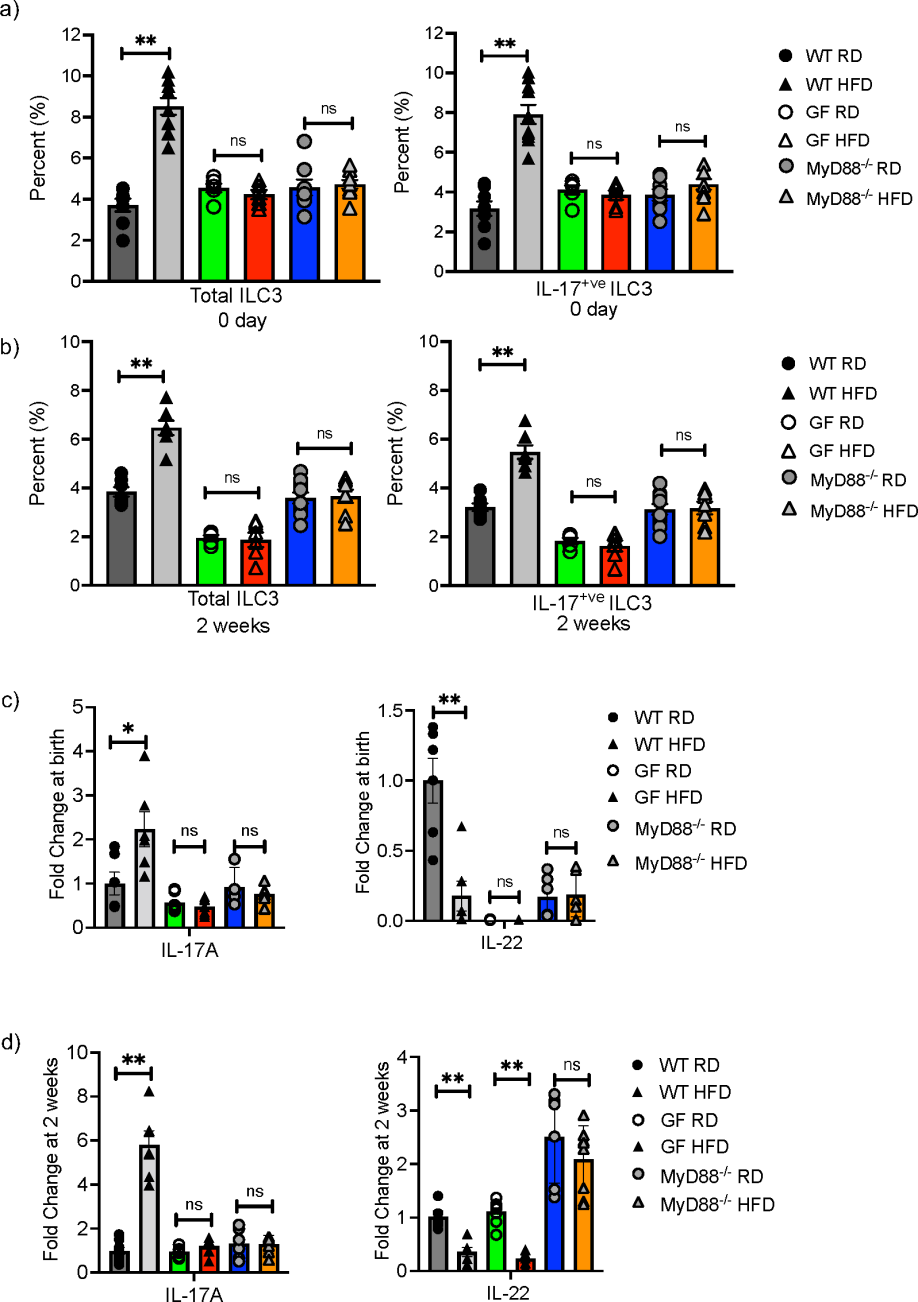
Germ-free and MyD88 deficient neonatal mice are protected from maternal HFD induced expansion of IL-17 producing ILC3. Flow cytometric quantification of total ILC3 and IL-17+ ILC3 in offspring born to RD and HFD exposed mothers from conventional (gray), germ-free (GF) (green and red bars) and MyD88-/- (Blue and orange bars) mice at birth and at 2-weeks of life. **(A)** IL-17 producing ILC3 were expanded at 0 days in conventional WT offspring born to mHFD dams. There was no difference in IL-17 producing ILC3 in mHFD exposed germ-free and MyD88 deficient offspring when compared to RD offspring at birth. **(B)** IL-17 producing ILC3 were expanded at 2-weeks old in conventional WT offspring born to mHFD dams. There was no difference in IL-17 producing ILC3 in mHFD exposed germ-free and MyD88 deficient offspring when compared to RD offspring at 2 weeks. **(C)** There was no difference in the expression of IL-17A or IL-22 analyzed by quantitative real time PCR (log scale) in small intestine from GF and MyD88 deficient mice at birth. **(D)** There was no difference in the expression of IL-17A in small intestine from GF and MyD88 deficient mice at 2 weeks old, but there was a decrease in IL-22 expression in GF mHFD offspring at 2 weeks old. The data shown are representative of 3 experiments with 6-10 mice in total for each group. Data are depicted as mean ± SEM with *p < 0.05 and **p < 0.01 according to two-way ANOVA. ns, not significant.

We next sought to determine if the microbiome played a role in the mHFD phenotype by examining germ-free (GF) and MyD88 deficient (MyD88^-/-^) mice on HFD and RD. In our previous study, we found that the maternal microbiome was unique when compared to the neonatal microbiome (2), however this does not preclude that the maternal microbiome interacts with the HFD diet to modify mucosal innate immunity in offspring. We therefore examined offspring at birth and 2-weeks of life and found that the percentage of total ILC3 and IL17^+ve^ ILC3 were similar in GF and MyD88^-/-^ RD and HFD offspring, unlike in conventional WT RD and HFD offspring (**Figures 3A, B**). There was no difference in IL-17A expression at birth (**Figure 3C**) and 2-weeks (**Figure 3D**), however there was an unexpected decrease in IL-22 mRNA in the GF HFD offspring at 2 weeks old. Taken together, these data strongly suggested that the microbiota plays a critical role in expansion of IL17^+ve^ ILC3 in mHFD offspring, potentially mediated by MyD88 signaling.

### Germ-free and MyD88 deficient mHFD offspring are protected from mHFD induced susceptibility to intestinal injury

**Figure 4:**
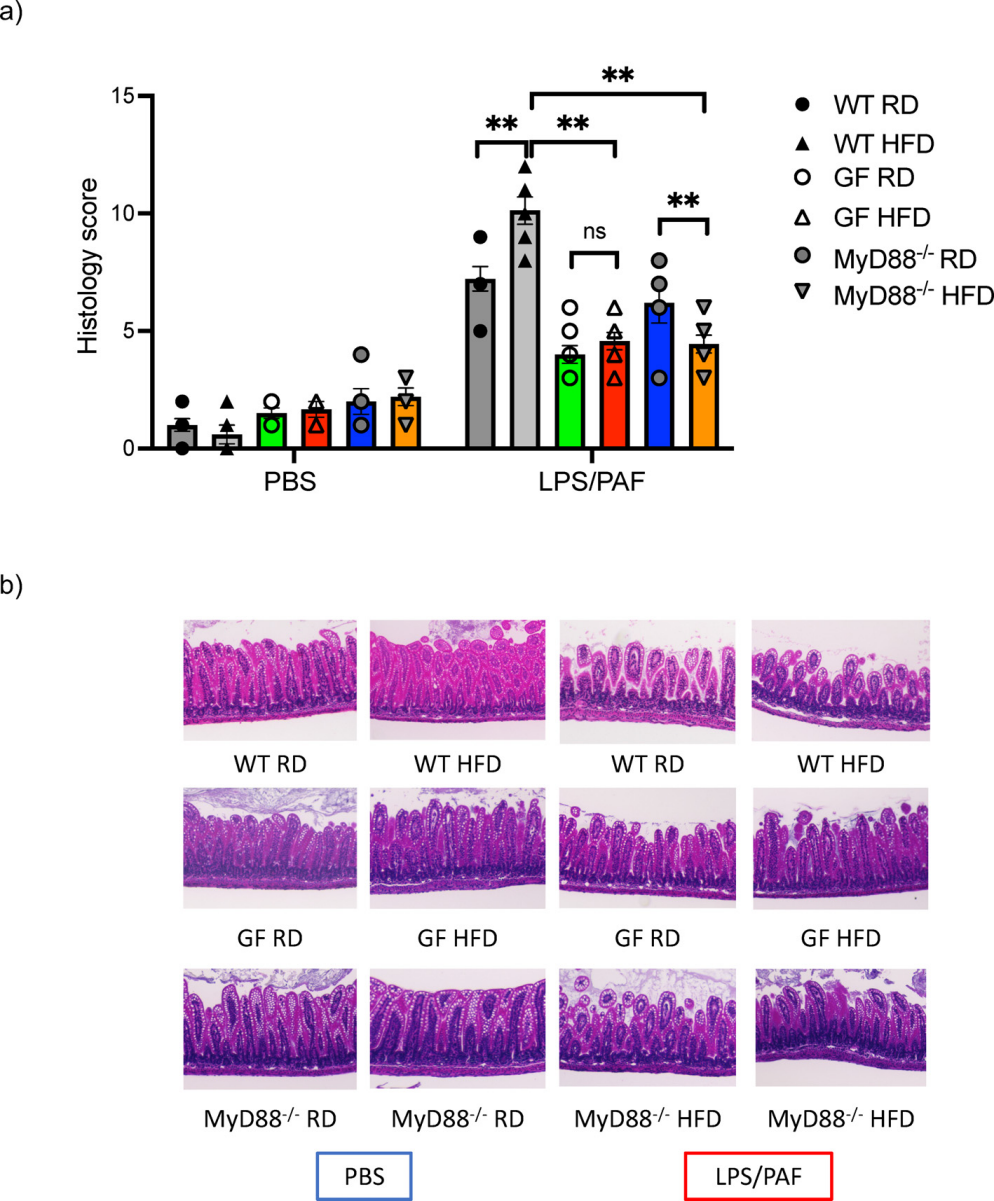
Germ-free and MyDD8^-/-^ mHFD offspring are protected intestinal injury after LPS/PAF exposure. **(A)** Quantification of histological injury score in RD and HFD offspring exposed to LPS/PAF in conventional (gray), GF (green and red bars) and MyDD^-/-^ (blue and orange bars) mice. **(B)** Representative image of H&E staining of small intestinal section in WT offspring exposed to LPS/PAF in RF and HFD offspring in conventional, germ-free and MyD88 deficient mice. Images displayed are using 10× magnification. The data shown are representative of 3 experiments with 4-8 mice in each group. Data are depicted as mean ± SEM with **p <0.01 according to two-way ANOVA. ns, not significant.

We next examined whether GF and MyD88 deficient offspring born to HFD mothers had similar susceptibility to intestinal inflammation as conventional WT mHFD offspring. We have previously demonstrated that WT and Rag deficient offspring born to mHFD dams had an increased susceptibility to an established model of necrotizing enterocolitis (NEC) which was partially blocked with IL-17A antibody, strongly suggesting that the injury is likely due to the expanded IL-17^+ve^ ILC3, and not Th-17 cells (2). We exposed GF and MyD88 deficient HFD and RD offspring to this established LPS/PAF (lipopolysaccharide and platelet-activating factor) model of intestinal injury. We found that RD and HFD GF offspring were protected from LPS/PAF injury and had similar susceptibility to intestinal injury, unlike in conventional HFD offspring who are highly susceptible to LPS/PAF injury (**Figures 4A, B**). Further, MyD88^-/-^ offspring born to HFD mothers were also protected from LPS/PAF injury (**Figures 4A, B**).

### Amniotic fluid metabolites are sufficient to expand ILC3 in GF neonatal mice

**Figure 5:**
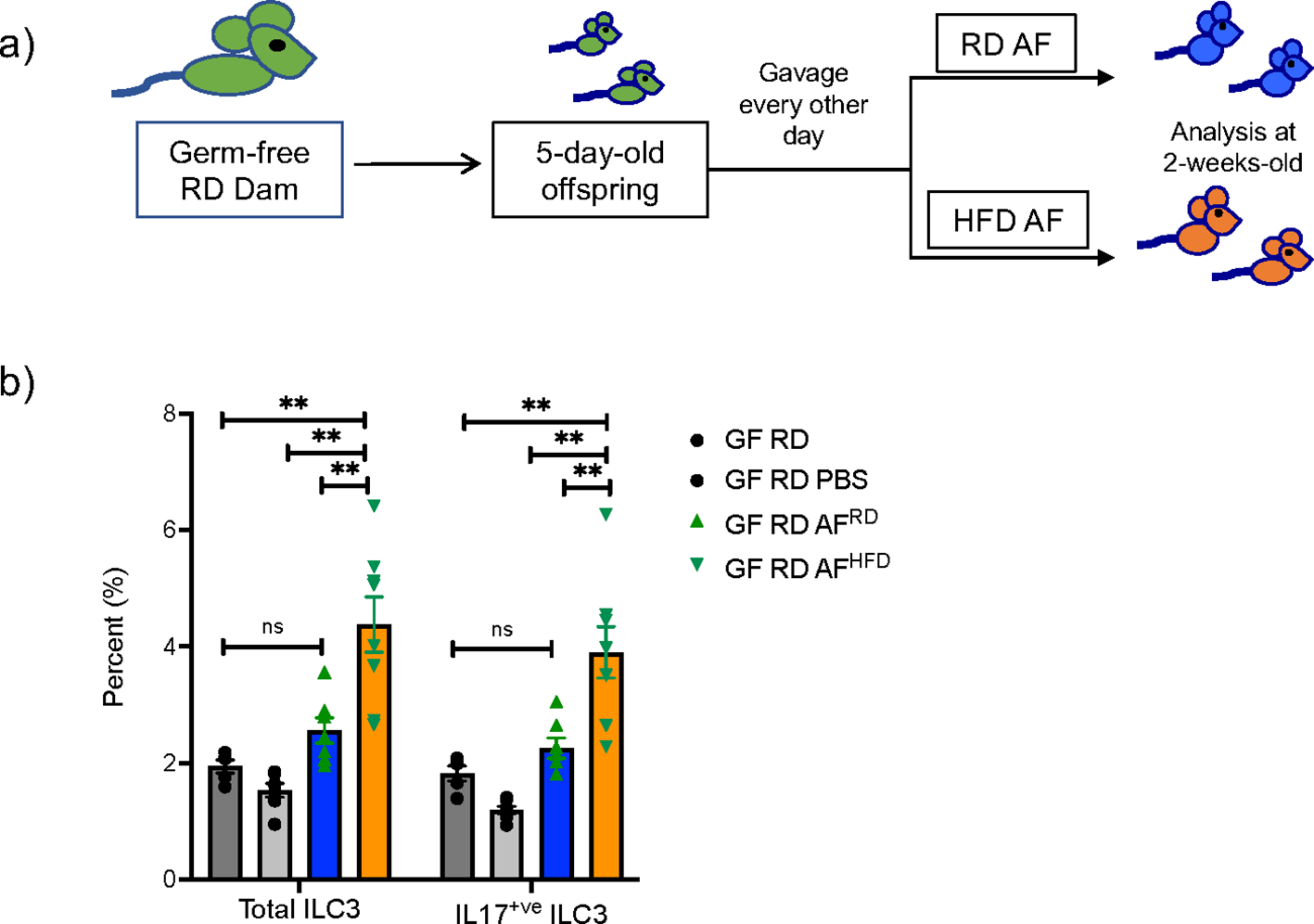
Amniotic fluid metabolites from conventional mHFD dams can expand ILC3 in germ-free neonatal mice. **(A)** Experimental design. Germ-free dams on regular diet were mated and offspring at 5-days of life were gavaged every other day with RD AF or HFD AF from conventional pregnant dams. Offspring were then examined at 2-weeks of life. **(B)** Flow cytometric quantification of total ILC3 and IL-17+ ILC3 in GF neonatal mice not gavaged with AF, gavaged with PBS, RD AF and HFD AF showing HFD AF expands total ILC3 and IL- 17+ ILC3. The data shown are representative of 3 experiments with a total of 4-6 mice in each group for germ- free gavage experiments. Data are depicted as mean ± SEM with way ANOVA. **p < 0.01 according to two-. ns, not significant.

AF is produced early in gestation primarily by ultra-filtration of the maternal plasma. The fetus swallows significant amounts of AF during gestation and the intestinal mucosa is consistently exposed to AF metabolites. We hypothesized that AF metabolites that are modified by mHFD could induce expansion of IL-17^+ve^ ILC3 *in utero* since ILC3 were expanded at birth. We performed timed mating of RD and HFD WT conventional and GF dams and collected AF at E15 (**Figure 1A**). To determine if the HFD AF could expand ILC3, we gavaged neonatal GF mice, and maintained them as germ-free using a strictly controlled germ-free isolator facility, with AF from conventional RD and HFD dams (**Figure 5A**), or PBS, for 10 days and found that at 2-weeks-old the HFD AF gavaged pups had an expansion of ILC3 and IL17^+ve^ ILC3 (**Figure 5B**), demonstrating that the mHFD AF was sufficient to expand IL17^+ve^ ILC3 in neonatal pups.

## Discussion

ILC3 are heterogenous mucosal innate immune cells that can produce both IL-17A and IL-22 (11). They are modulated by dietary metabolites, including indole derivatives and adaptive immune cells (28, 29). IL-17A is a cytokine involved in intestinal inflammation and is increased in neonatal necrotizing enterocolitis in humans and in murine models (11). We have previously demonstrated that mHFD exposure increases susceptibility to intestinal inflammation mediated by an increase in IL-17^+ve^ ILC3 (2). In this study, we observed that expansion of Firmicutes and IL17^+ve^ ILC3 in mHFD offspring is present in mice born via C-section and vaginal delivery, suggesting a prenatal effect that is not solely driven by transfer of the maternal microbiome via the vaginal canal during birth. Instead, it is likely that the maternal gastrointestinal microbiome plays an important role in directing ILC3 in offspring. We further found that germ-free mice and MyD88 deficient mice are protected from the mHFD phenotype, demonstrating a role for the maternal microbiome and MyD88. We show that even at birth, ILC3 are expanded in WT mHFD offspring, but not in germ-free or MyD88 deficient offspring. Finally, we identify a novel mechanism for maternal-diet-induced expansion of ILC3 in the fetus, mediated by interaction of the maternal microbiome and maternal diet, to modulate amniotic fluid metabolites and expand IL17^+ve^ ILC3.

It is interesting to note that while IL17^+ve^ ILC3 are primarily increased in mHFD offspring during the neonatal period, we found they are also expanded at birth. While other cell types, particularly Th17 cells, can produce IL-17A, we have previously shown, utilizing Rag deficient mice, that the phenotype is primarily due to expansion of IL17^+ve^ ILC3 in mHFD offspring (2). This suggests that there is pre- and post-natal regulation of expansion, with the prenatal component being dependent on maternal diet and the maternal microbiome. It is possible that modulation of innate immunity post-natally is dependent on loss of cells through apoptosis or pryoptosis (30), via microbiota mediated signaling. The neonatal microbiome and toll-like receptor (TLR) signaling may play a role in maintaining IL17^+ve^ ILC3 in mHFD offspring or microbial mediated destruction of IL17^+ve^ ILC3 in RD offspring.

Not surprisingly, we found that germ-free offspring were protected from the mHFD phenotype. Germ-free mice are also known to be protected from HFD-induced obesity and the breakdown of fat and energy harvesting by the microbiome are important in the development of obesity (31), in addition to calorie and fat content. One mechanism for mHFD effects on the offspring includes differential breakdown of the diet by the maternal microbiome which subsequently modifies delivery of nutrients to offspring. Another possible mechanism, suggested by our study, is by diet-induced microbial modification of metabolites within amniotic fluid which then act to regulate immunity in the fetus and subsequently after birth. Since diet can alter the metabolites present in the serum (32, 33), and AF is produced by ultrafiltration of the maternal serum early in pregnancy, the potential for early priming of the immune response by diet-modulated metabolites is intriguing. Future studies aimed at examining the role of specific microbial profiles on AF composition by utilizing conventionalized mice with the maternal and neonatal HFD microbiome would also be important. Targeted microbiome colonization experiments would provide more insight into the specific microbes that are responsible for the differential metabolites seen in HFD AF. It is notable that later in gestation the fetus contributes to the AF via lung secretions and urine, and this study is limited in differentiating whether maternal or fetal components of the AF are critical for the phenotype.

The timing of the effect from maternal high fat diet exposure is also notable since the AF metabolomics analysis was performed in AF obtained at the first pregnancy after the dam was only exposed to the HFD for 4 weeks and during pregnancy. This short time period was sufficient to modify the metabolite composition in amniotic fluid. The model also highlights the effect of the maternal diet, and not obesity, since the mHFD exposure is limited and the dams do not develop obesity as seen in ob/ob dams, which are usually 60-70g (**Supplementary Figure 1**).

Indeed, the fact that the AF metabolites alter immune function post-natally as well, as demonstrated by the germ-free AF gavage experiment, suggests that priming of the offspring immune system is not just limited to the *in utero* period but can be extended to the neonatal period and beyond. Germ-free mice were examined since they have a naïve intestinal environment not modified or influenced by metabolites produced by bacteria. While we did not show that HFD AF gavage can also increase LPS/PAF injury and we didn’t examine MyD88 deficient germ-free mice, we speculate that HFD AF gavage would increase LPS/PAF injury and MyD88 deficiency would be protective. Future studies aimed at examining if these metabolites can modify innate immunity in the colonized intestinal environment will also be informative. While we utilized HFD and RD AF for the gavage experiment, further studies on specific metabolites or combination of metabolites in the colonized and germ-free environment will be important to determine which metabolites are causative. This opens a novel direction for the modulation of immunity in preterm and newborn infants via development of metabolite supplements to prevent or treat disease in this vulnerable population.

Another key finding of this study is the need for MyD88 signaling in the mHFD phenotype in offspring. This suggests that maternal diet mediated signaling is via toll-like receptor (TLR) signaling or that TLR signaling in the fetus is critical for modulating immunity *in utero*. Several studies have demonstrated that dietary metabolite modulation of immunity is dependent on TLR (34) or other xenobiotic sensors (35), however this requires further investigation. Metabolites may interact with TLRs and this mechanism may also be responsible for the loss of the HFD phenotype seen in MyD88 deficient offspring. Further studies to differentiate whether maternal or fetal TLR signaling is also critical for the maternal diet induced phenotype.

In conclusion, we have demonstrated that maternal HFD effects on the intestinal ILC3 population in offspring are independent on the mode of delivery and are dependent on the maternal microbiome and MyD88 signaling. We show that mHFD exposure modifies metabolites within the amniotic fluid compartment and HFD AF can differentially prime the fetal intestinal immune system by expanding IL17^+ve^ ILC3. The results of this study provides a novel mechanism of maternal diet mediated effects on the offspring and provides potential new targets for preventative and therapeutic strategies in preterm babies.

## Data Availability

The original contributions presented in the study are included in the article/**Supplementary Material**, further inquiries can be directed to the corresponding author/s.

## References

[1] Gawlinska K Gawlinski D Filip M Przegalinski E. Relationship of maternal high-fat diet during pregnancy and lactation to offspring health. Nutr Rev. (2021) 79:709–25. doi: 10.1093/nutrit/nuaa020 32447401

[2] Babu ST Niu X Raetz M Savani RC Hooper LV Mirpuri J. Maternal high-fat diet results in microbiota-dependent expansion of ILC3s in mice offspring. JCI Insight. (2018) 3(19):e99223. doi: 10.1172/jci.insight.99223 30282818 PMC6237468

[3] Guibourdenche M El Khayat El Sabbouri H Djekkoun N Khorsi-Cauet H Bach V Anton PM. Programming of intestinal homeostasis in male rat offspring after maternal exposure to chlorpyrifos and/or to a high fat diet. Sci Rep. (2021) 11:11420. doi: 10.1038/s41598-021-90981-2 34075131 PMC8169651

[4] Myles IA Fontecilla NM Janelsins BM Vithayathil PJ Segre JA Datta SK. Parental dietary fat intake alters offspring microbiome and immunity. J Immunol. (2013) 191:3200–9. doi: 10.4049/jimmunol.1301057 PMC3831371 23935191

[5] Andreas E Reid M Zhang W Moley KH. The effect of maternal high-fat/high-sugar diet on offspring oocytes and early embryo development. Mol Hum Reprod. (2019) 25:717–28. doi: 10.1093/molehr/gaz049 PMC6884416 31588490

[6] Sasson IE Vitins AP Mainigi MA Moley KH Simmons RA. Pre-gestational vs gestational exposure to maternal obesity differentially programs the offspring in mice. Diabetologia. (2015) 58:615–24. doi: 10.1007/s00125-014-3466-7 PMC4452998 25608625

[7] Ribaroff GA Wastnedge E Drake AJ Sharpe RM Chambers TJG. Animal models of maternal high fat diet exposure and effects on metabolism in offspring: a meta-regression analysis. Obes Rev. (2017) 18:673–86. doi: 10.1111/obr.12524 PMC5434919 28371083

[8] Bordeleau M Comin CH Fernandez de Cossio L Lacabanne C Freitas-Andrade M Gonzalez Ibanez F. Maternal high-fat diet in mice induces cerebrovascular, microglial and long-term behavioural alterations in offspring. Commun Biol. (2022) 5:26. doi: 10.1038/s42003-021-02947-9 35017640 PMC8752761

[9] Di Gesu CM Matz LM Bolding IJ Fultz R Hoffman KL Gammazza AM. Maternal gut microbiota mediate intergenerational effects of high-fat diet on descendant social behavior. Cell Rep. (2022) 41:111461. doi: 10.1016/j.celrep.2022.111461 36223744 PMC9597666

[10] Shapiro ALB Ringham BM Glueck DH Norris JM Barbour LA Friedman JE. Infant Adiposity is Independently Associated with a Maternal High Fat Diet but not Related to Niacin Intake: The Healthy Start Study. Matern Child Health J. (2017) 21:1662–8. doi: 10.1007/s10995-016-2258-8 PMC5517356 28161859

[11] Mirpuri J. The emerging role of group 3 innate lymphoid cells in the neonate: interaction with the maternal and neonatal microbiome. Oxf Open Immunol. (2021) 2:iqab009. doi: 10.1093/oxfimm/iqab009 34151271 PMC8208228

[12] WHO. Obesity and overweight. (2017).

[13] Hales CM Carroll MD Fryar CD Ogden CL. Prevalence of obesity and severe obesity among adults: United States, 2017-2018. NCHS Data Brief. (2020) (360):1–8. 32487284

[14] Denizli M Capitano ML Kua KL. Maternal obesity and the impact of associated early-life inflammation on long-term health of offspring. Front Cell Infect Microbiol. (2022) 12:940937. doi: 10.3389/fcimb.2022.940937 36189369 PMC9523142

[15] Mirpuri J. Evidence for maternal diet-mediated effects on the offspring microbiome and immunity: implications for public health initiatives. Pediatr Res. (2021) 89:301–6. doi: 10.1038/s41390-020-01121-x PMC7897208 32919391

[16] Grant ET Boudaud M Muller A Macpherson AJ Desai MS. Maternal diet and gut microbiome composition modulate early-life immune development. EMBO Mol Med. (2023) 15:e17241. doi: 10.15252/emmm.202217241 37278126 PMC10405054

[17] Sikder MAA Rashid RB Ahmed T Sebina I Howard DR Ullah MA. Maternal diet modulates the infant microbiome and intestinal Flt3L necessary for dendritic cell development and immunity to respiratory infection. Immunity. (2023) 56:1098–114 e10. doi: 10.1016/j.immuni.2023.03.002 37003256

[18] Fernandes KA Lim AI. Maternal-driven immune education in offspring. Immunol Rev. (2024) 323:288–302. doi: 10.1111/imr.v323.1 38445769

[19] Everard A Geurts L Caesar R Van Hul M Matamoros S Duparc T. Intestinal epithelial MyD88 is a sensor switching host metabolism towards obesity according to nutritional status. Nat Commun. (2014) 5:5648. doi: 10.1038/ncomms6648 25476696 PMC4268705

[20] Liu Z Sun H Xu S Wang H Zhang Z Wei Y. Dietary ingredient change induces a transient MyD88-dependent mucosal enteric glial cell response and promotes obesity. Nutr Neurosci. (2022) 26(12):1183–1193. doi: 10.1080/1028415X.2022.2142129 36342063

[21] Shuai W Kong B Fu H Shen C Huang H. Loss of MD1 increases vulnerability to ventricular arrhythmia in diet-induced obesity mice via enhanced activation of the TLR4/MyD88/CaMKII signaling pathway. Nutr Metab Cardiovasc Dis. (2019) 29:991–8. doi: 10.1016/j.numecd.2019.06.004 31353205

[22] Brace RA. Physiology of amniotic fluid volume regulation. Clin Obstet Gynecol. (1997) 40:280–9. doi: 10.1097/00003081-199706000-00005 9199840

[23] Zhou Y Zhao R Lyu Y Shi H Ye W Tan Y. Serum and amniotic fluid metabolic profile changes in response to gestational diabetes mellitus and the association with maternal-fetal outcomes. Nutrients. (2021) 13(10):3644. doi: 10.3390/nu13103644 34684645 PMC8539410

[24] Gomez de Aguero M Ganal-Vonarburg SC Fuhrer T Rupp S Uchimura Y Li H. The maternal microbiota drives early postnatal innate immune development. Science. (2016) 351:1296–302. doi: 10.1126/science.aad2571 26989247

[25] Hooper LV Mills JC Roth KA Stappenbeck TS Wong MH Gordon JI. Molecular Cellular Microbiology. Sansonetti P Zychlinsky A, editors. San Diego: Academic (2002).

[26] Mirpuri J Sotnikov I Myers L Denning TL Yarovinsky F Parkos CA. Lactobacillus rhamnosus (LGG) regulates IL-10 signaling in the developing murine colon through upregulation of the IL-10R2 receptor subunit. PloS One. (2012) 7:e51955. doi: 10.1371/journal.pone.0051955 23272193 PMC3525658

[27] Sridharan GV Choi K Klemashevich C Wu C Prabakaran D Pan LB. Prediction and quantification of bioactive microbiota metabolites in the mouse gut. Nat Commun. (2014) 5:5492. doi: 10.1038/ncomms6492 25411059

[28] Artis D Spits H. The biology of innate lymphoid cells. Nature. (2015) 517:293–301. doi: 10.1038/nature14189 25592534

[29] Panda SK Colonna M. Innate lymphoid cells in mucosal immunity. Front Immunol. (2019) 10:861. doi: 10.3389/fimmu.2019.00861 31134050 PMC6515929

[30] Paludan SR Pradeu T Masters SL Mogensen TH. Constitutive immune mechanisms: mediators of host defence and immune regulation. Nat Rev Immunol. (2021) 21:137–50. doi: 10.1038/s41577-020-0391-5 PMC7418297 32782357

[31] Backhed F Manchester JK Semenkovich CF Gordon JI. Mechanisms underlying the resistance to diet-induced obesity in germ-free mice. Proc Natl Acad Sci U S A. (2007) 104:979–84. doi: 10.1073/pnas.0605374104 PMC1764762 17210919

[32] Acar E Gurdeniz G Khakimov B Savorani F Korndal SK Larsen TM. Biomarkers of individual foods, and separation of diets using untargeted LC-MS-based plasma metabolomics in a randomized controlled trial. Mol Nutr Food Res. (2019) 63:e1800215. doi: 10.1002/mnfr.201800215 30094970

[33] Pigsborg K Gurdeniz G Rangel-Huerta OD Holven KB Dragsted LO Ulven SM. Effects of changing from a diet with saturated fat to a diet with n-6 polyunsaturated fat on the serum metabolome in relation to cardiovascular disease risk factors. Eur J Nutr. (2022) 61:2079–89. doi: 10.1007/s00394-021-02796-6 PMC9106625 34999928

[34] Fessler MB Rudel LL Brown JM. Toll-like receptor signaling links dietary fatty acids to the metabolic syndrome. Curr Opin Lipidol. (2009) 20:379–85. doi: 10.1097/MOL.0b013e32832fa5c4 PMC3099529 19625959

[35] Venkatesh M Mukherjee S Wang H Li H Sun K Benechet AP. Symbiotic bacterial metabolites regulate gastrointestinal barrier function via the xenobiotic sensor PXR and Toll-like receptor 4. Immunity. (2014) 41:296–310. doi: 10.1016/j.immuni.2014.06.014 25065623 PMC4142105

