# Supplementary material for: Maternal high fat diet exposure modifies amniotic fluid metabolites and expands group 3 innate lymphoid cells dependent on the maternal microbiome and MyD88-signaling
Source: Front Immunol. 2024 Nov 18;15:1439804. doi: 10.3389/fimmu.2024.1439804 (PMC11609847; doi:10.3389/fimmu.2024.1439804)
Supplement: Supplementary file 1 [file DataSheet1.pdf]

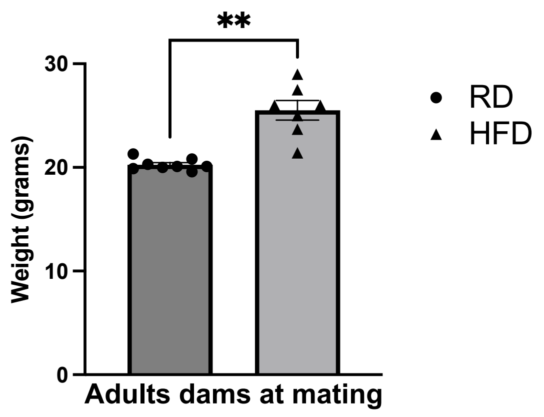

Supplementary Figure 1: **Average weight of breeding regular diet (RD) and high fat diet (HFD) dams.** HFD dams had increased weight compared to RD dams but were not obese.

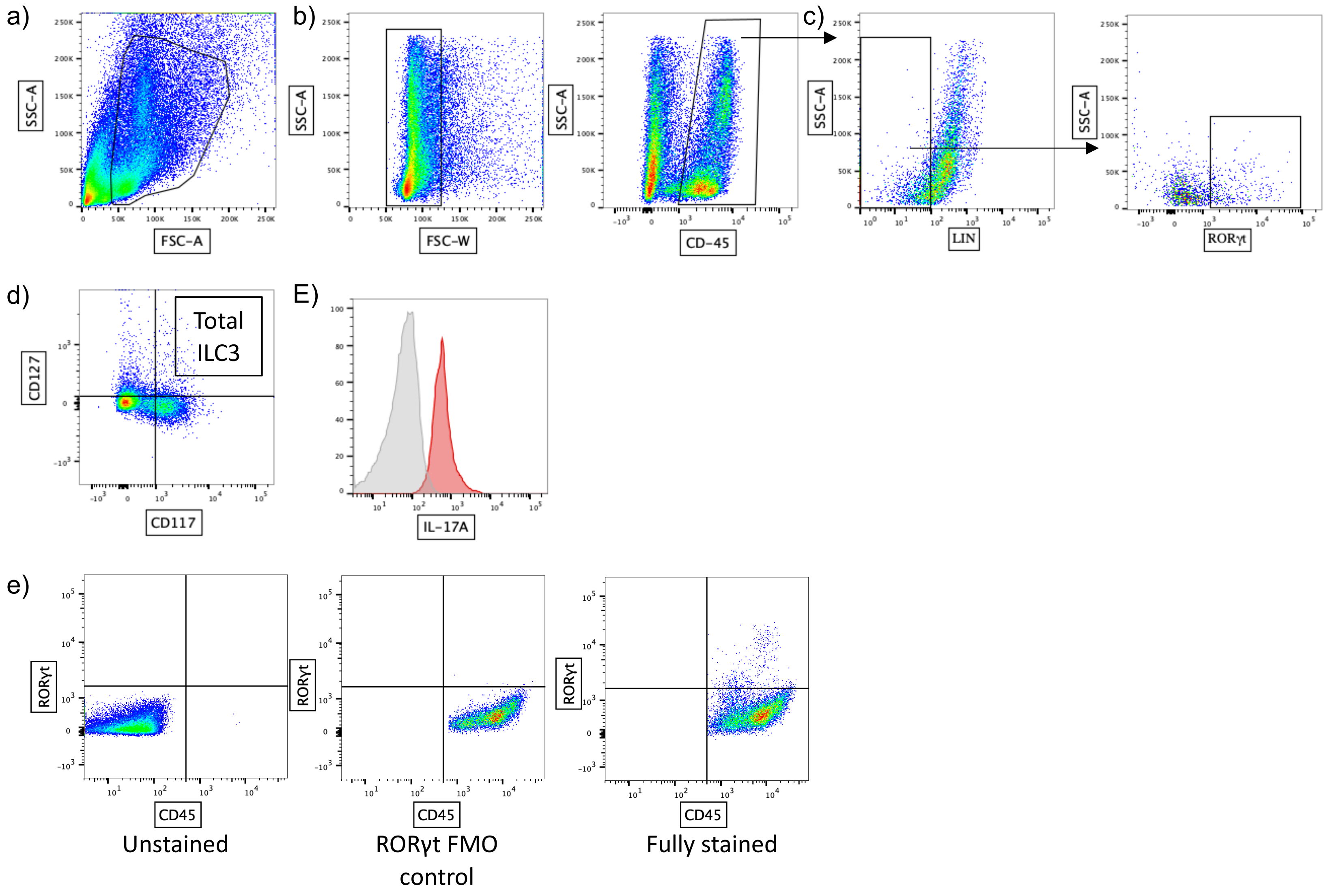

Supplementary Figure 2: **Gating strategy for group 3 innate lymphoid cells from murine neonatal small intestine.** A) a) Lymphocytes were gated using forward/side scatter (FSC/SSC). b) Single cells and CD45+ve cells were gated from lymphocytes. c) CD45+ve population was further gated for LIN negative and RORγt positive to distinguish ILC3 cells. d) CD45+ve cells were gated for CD127 and CD117 positive cells and quantified as total ILC3. e) IL-17 positive cells were quantified (red histogram) compared to control (gray histogram) and expressed as percentage of total CD45 positive cells. f) Fluorescence minus one (FMO) staining for RORγt used for gating with panels representing unstained control, RORγt FMO control and fully stained.

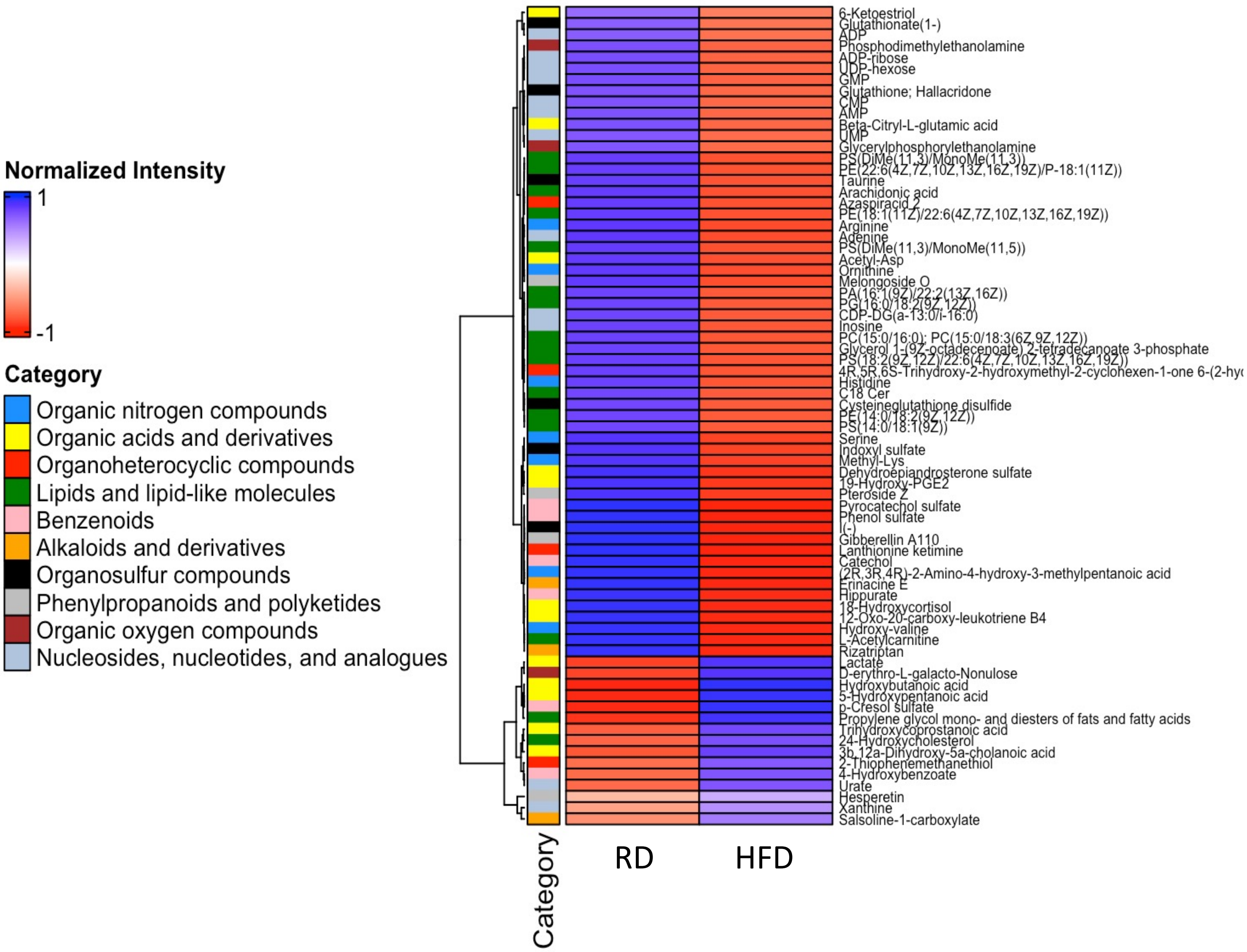

Supplementary Figure 3: **Amniotic fluid from high fat diet dams have uniquely modified metabolites.** Heat map represents normalized, averaged differentially expressed metabolites present in amniotic fluid from regular diet (RD) dams and high fat diet (HFD) dams. Data is representative of amniotic fluid samples from 3 dams with 8 individual sacs in the HFD group and 8 individual sacs in the RD group. The Category column represents the classification of each metabolite.

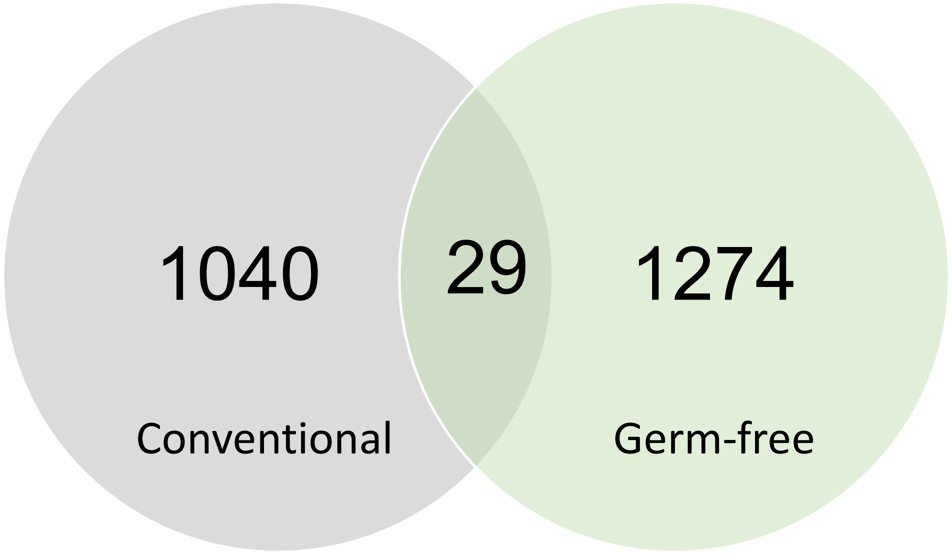

Supplementary Figure 4: **VENN diagram of overlapping metabolites between conventional and germ-free amniotic fluid.** There was minimal overlap of metabolites between germ-free and conventional mice in amniotic fluid. Data representative of all metabolites detected from conventional AF (16 samples) compared to germ-free AF (21 samples) in both RD and HFD conditions.

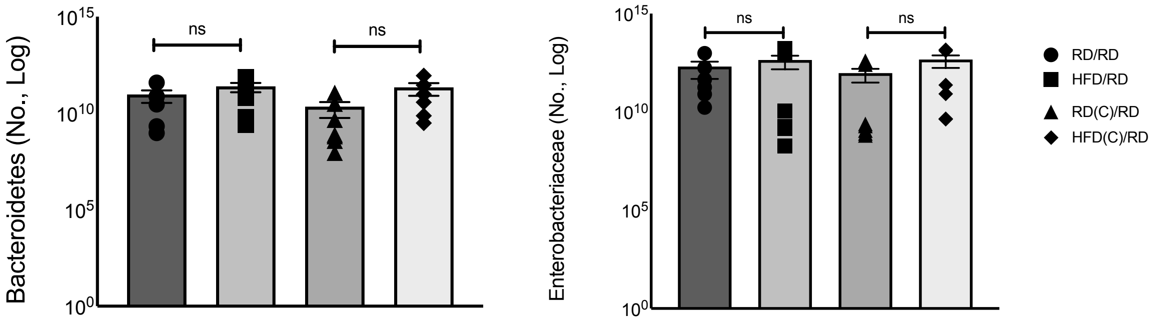

Supplementary Figure 5: **Quantification of Bacteroidetes and Enterobacteriaceae in colonic fecal samples from RD and HFD offspring born vaginally or via C-section**. There was no difference in these major bacterial phyla by qRT-PCR quantification. Data shown are representative of 5-6 mice in each group. Data are depicted as mean +/- SEM with * $p < 0.05$ and ** $p < 0.01$ according to one-way ANOVA with post-hoc Tukey's test.
